# Importance of pharmacist-patient relationship in people living with HIV and concomitant opioid use disorder

**DOI:** 10.1016/j.rcsop.2021.100052

**Published:** 2021-07-31

**Authors:** Alina Cernasev, Michael P. Veve, Kenneth C. Hohmeier, Nathan A. Summers, Santosh Kumar

**Affiliations:** a Department of Clinical Pharmacy and Translational Science, University of Tennessee Health Sciences Center, Nashville, TN, USA; b Department of Pharmacy Practice, Eugene Applebaum College of Pharmacy and Health Sciences, Wayne State University, 259 Mack Avenue, Detroit, MI 48201, USA; c Division of Infectious Diseases, Department of Medicine, University of Tennessee Health Science Center, Memphis, TN 38103, USA; d Department of Pharmaceutical Sciences, University of Tennessee Health Science Center, Memphis, TN, USA

**Keywords:** PLWHA, Opioids, OUD, Addiction, Pharmacist, Relationship

## Abstract

**Background:**

PLWHA commonly suffer from chronic pain that is often treated with opioids, leading to subsequent opiate use disorders. As the majority of Americans live in close proximity to a pharmacy, community pharmacists are well equipped to interact with PLWHA. Few data quantify the needs of PLWHA with OUD, or how they utilize community pharmacists.

**Objectives:**

To characterize the perceptions of Persons Living with HIV/AIDS (PLWHA) and using opioid medications on the interaction with pharmacists.

**Methods:**

For this study, a qualitative approach was used. A key purpose of interviews is to encourage and inspire the subject to share a significant event of his/her life with the interviewer. Recruitment for face-to-face interviews with PLWHA continued until saturation was achieved. The Theory of Planned Behavior was used to assess the findings from this study. Sixteen interviews were transcribed verbatim and content analysis was performed by two researchers using Dedoose®, a qualitative software. Codes were grouped based on similarities into categories that facilitated the emergence of themes.

**Results:**

Content analysis revealed two major themes. The first theme presents the subjects' beliefs and opinions about pharmacists' interactions when picking up their opioid prescriptions. Several subjects described encounters with the pharmacists that facilitated a trustful relationship. In the second theme, the analysis showed that the relationship with community pharmacists could be improved by having a more in-depth counseling about opioid medication and abuse.

**Conclusions:**

These data highlight how PLWHA would like to interact with pharmacists when picking up opioid prescriptions. These results depicted how some subjects are using the pharmacists as a vital resource for medication information. These findings also demonstrated how for some subjects a more detailed counseling session when they receive opioid medications could be crucial in changing their behavior. Thus, community pharmacists are well positioned to reduce the usage of opioid medications and change PLWHA behavior and attitudes toward opioid prescriptions.

## 1 Introduction

People living with HIV/AIDS (PLWHA) are at high risk for poor outcomes when using chronic opioids, which can include clinically significant drug-drug interactions, decreased antiretroviral (ARV) adherence, or lead to other recreational substance use that may contribute to morbidity and reduced survival.^1^, ^2^, ^3^ It is estimated that 21–95% of PLWHA are prescribed opioids for chronic pain management,^4^ which can subsequently progress to opioid use disorder (OUD) or other substance misuse. Pharmacists are key members of the healthcare team and can improve PLWHA outcomes through ARV selection and optimization, drug counseling, and monitoring.^1^ However, the role of pharmacists in monitoring opioid use, OUD management, and opioid education for PWLHA is relatively unknown.

The high prevalence of chronic pain observed in PLWHA and associated long-term opioid use can create serious individual patient and public health issues, such as injection drug use with unintentional disease spread or outbreak.^5^ While medications to treat OUD and other opioid rehabilitative services exist, healthcare disparities and access to addiction specialists disproportionally impact underserved populations like PLWHA.^3^ Community pharmacists are highly accessible healthcare providers that have the opportunity to educate and potentially mitigate OUD or prevent opioid misuse in at risk individuals, however, pharmacists may be an underutilized resource in PLWHA.^6^^,^^7^ The American Society of Health System Pharmacist Guidelines on Pharmacist Involvement in HIV Care suggest pharmacists should be active in screening for substance use disorders and collaborate with additional specialists.^1^ However, pharmacists currently serving in these roles are traditionally those who practice in ambulatory care clinic settings, and not in more highly accessible community-based pharmacies. Some data suggest a need for greater pharmacist involvement in the management of PLWHA and OUD, but have identified community pharmacists may not be well-prepared to engage in opioid misuse-related patient conversations.^4^^,^^8^ However, there are essentially no studies identifying the perspective of PLWHA who use opioids and their perception regarding the role of community pharmacists within their health management.

The purpose of this study was to evaluate PLWHA beliefs related to their experiences with community pharmacists and use of opioids with ARV medications**.** There are limited qualitative analyses describing the value and importance of the pharmacist-patient relationship in PLWHA with OUD, particularly from the perspective of the patient. The results from this study will provide insight in how community pharmacists can improve the quality of care in PLWHA who use opioids.

## 2 Methods

A face-to-face interview using the Theory of Planned Behavior (TPB) as the conceptual framework aimed to characterize the predictors of their perceived attitude, subjective norm, and control toward any behavior.^9^ TPB uses a synergy between a person's behavioral beliefs, normative beliefs, and control beliefs as predictors of their perceived attitude, subjective norm, and control toward any behavior.^9^ TPB has been applied to various areas of research including vaccination, HIV/AIDS prevention, smoking cessation, medication therapy management, and medication administration.^10^, ^11^, ^12^, ^13^

### 2.1 Participants and data collection

The study received approval from the Institutional Review Board (IRB) of the University of Tennessee Health Science Center (#19–07002-XM). Subjects were recruited via fliers from HIV clinics and AIDS service organization located in three distinct regions of Tennessee: Nashville, Memphis, and Knoxville. The inclusion criteria were adult PLWHIV who identified as present or past users of opioid medications, English speakers, and willingness to share their experiences when using pharmacy services. The interview guide consisted of open-ended questions using TPB developed by the research team (clinical pharmacists and physicians) with experience in HIV and OUD to elicit subjects' experiences with pharmacists and usage of opioids and ARV medications. Verbal consent was obtained prior to each interview, and the respondents agreed to be audio recorded. Each transcript was transcribed verbatim by a third party to avoid any biases. To ensure subject privacy, demographic information was not collected; however, some subject voluntarily disclosed their marital status, the number of years since they were diagnosed with HIV, or if they have been taking opioid medications.

The transcripts were imported into Dedoose® (CA) qualitative software, where deductive and inductive content analyses informed by TPB was performed.^14^ To ensure rigor for the data analysis, Lincoln and Guba's framework were used.^15^ For example, two researchers (AC, KH) separately read all the transcripts and coded all the data until a final coding frame was obtained.^15^ The researchers then met and discussed any differences between the codes and categories and finalized the final themes based on the similarities of the categories. To ensure that thematic saturation was achieved, the research team met and discussed each transcript and determined if additional interviews were necessary.^16^^,^^17^ Furthermore, auditability was maintained by documenting all memos relevant to interpretations and decisions regarding thematic analysis during data analysis.^15^ This process was facilitated by using Dedoose® software.^15^ Credibility was achieved throughout all the stages of data collection and data analysis.^15^ Subsequently, the consolidated criteria for reporting qualitative research (COREQ) was consulted throughout the data collection, analysis, and interpretation.^18^

## 3 Results

Sixteen interviews were conducted between December 2019 and August 2020. Each interview was approximately 40–120 min in length. The majority of subjects identified themselves as men (*n* = 13) and African American (*n* = 14). Two subjects stated that they were illiterate (i.e., could not write or read). Subjects stated that they were taking ARV medications at the time of the interview. Most subjects (*n* = 12) stated that they used opioids or were still taking opioid prescriptions at the time of the interview. All subjects were insured through Medicaid or through the Health Resources and Service Administration Ryan White HIV/AIDS program, which is a program dedicated to the comprehensive medical care of PLWHA in the United States.^19^

### 3.1 Theme 1: The patient-pharmacist relationship is valued: “…*they're looking out for their patient, like a doctor would”*

This theme depicts the subjects' beliefs and opinions about pharmacists' interactions when picking up their prescriptions. Core to this theme was the value subjects placed on an established relationship between patient and pharmacist. Although subjects noted the clinical expertise the community pharmacist possessed, it was the trustful relationship with the pharmacist and their efforts in navigating the complex healthcare system that patients valued highest.

Frequently, subjects felt that the pharmacist's relationship was strong because that pharmacist was familiar with their name or talked to them in a manner that fostered trust. Subject 4 presents the relationship with that pharmacy:

*“ …a relationship… Oh, yeah, they remember me. They say, hey Mr. [NAME]. Mr. so-and-so, how you doing today? I say, I'm here to pick up my meds. Oh, we got them for you. Hold on for a few minutes. And they go back and get them [the antiretroviral prescription]…or I might crack a joke or something then, you know, the person laughs. And here you go Mr. [NAME]…”* (S4, Male).

One subject highlighted the importance of receiving phone calls about his wellbeing. In this situation, the subject would engage in a conversation with the pharmacy staff that would benefit him. It is important to note that this subject is illiterate and stated throughout the interview that sometimes he would be depressed. “They made my day” has echoes of the most significant effect on this person. This subject also felt the pharmacist cared about him personally. He reported that their phone conversations would alleviate his external dark thoughts and feelings of depression.

*“…they call me and ask me if I'm doing all right because sometimes, I might be depressed, and that phone call right there just done it…they made my day!”*

*INTERVIEWER: So the phone call makes you happy?*

*Respondent: Very, very happy….I am not depressed after that phone call…”* (S3, Male).

A number of subjects pointed to a problematic issue being the lack of insurance coverage for ARV medications. For example, S16 feels a deep and caring relationship between him and the pharmacist. S16 believes that the pharmacist went above and beyond to ensure he would receive his ARV that keeps him alive. One can see how this experience with the pharmacist would contribute to a sense of faith in the profession. As a result of the pharmacist's actions, S16 explicitly thinks the pharmacist is “*looking out for their patient, like a doctor would.”*

*“They're just- friendly and open, and one time, it was near the end of a year with an insurance company, and I had one pharmacist, somehow, it was 30-day supplies, but he was concerned that- what would happen the following year, and he actually got the insurance company, and he pushed the most expensive medicine and got them to approve a three-month supply to be assured I would have the higher dollar one.”* (S16, Male).

### 3.2 Theme 2: Patient-pharmacist communication needs:” *…if [pharmacist] have told me that it was a matter of life and death…then…I would've not done what I did do…”*

This theme centers on the subjects' stories about addiction and their interaction with pharmacists when picking up a prescription opioid for chronic pain. Perceptions of communication were positive, but subjects also made explicit recommendations to improve the quality of pharmacist-patient communication.

Although the subjects acknowledged the use of prescription opioids for diagnosed pain conditions, several felt unable to be open about any misuse during the interviews. For example, the subjects did not want to elaborate the reasons why they misused the opioid treatment. Furthermore, a few subjects mentioned that they were incarcerated and preferred to keep private the information. Several subjects described those days when they were started on opioid medications for their chronic pain as unaware of possible misuse consequences. For S13, described as “being trapped” in this situation for years, she is constantly “searching for pain medications.” S13 points out how the status became habitual, and sometimes she purchases the pain medications “from the street” to deal with the pain. S10 highlights that he is not well, and withdrawal triggers him to take his pain medications. During the interviews the subjects described the desire to decrease the amount of pain medications that would allow them to have a “normal” life again. Some subjects highlighted what type of counseling they would have preferred to hear from the pharmacist when picking up opioid prescriptions. S14 powerfully summarizes the message he would have liked to hear from his pharmacist when he started taking pain medications:

*“…if [ pharmacist] have told me that it was a matter of life and death, then I would've been happy. I would've not done what I did do. If [pharmacist] had used different language I wouldn't have gone to prison…”.*

Subjects felt that pharmacists could use less ambiguous and more serious words and warn them about the consequences of opioid misuse, which would have given them an opportunity to decide whether to take the opioid prescription. The below extract demonstrates the value of receiving counseling. More detailed counseling at the initial prescription is recommended by the subject.

*“…one [pharmacist] that kind a knew a little about me, about what I was getting, and he used to tell me all the time, man… be careful with this medicine. Kept on telling me every time I come, “Be careful, be careful,“ but I couldn't figure him out. I always ask, “Why?“ and he said, “I just- I can just tell you, be careful.”* (S9, Male).

## 4 Discussion

This qualitative study includes the novel finding that subjects are interested in learning from pharmacists about how addiction occurs and provided various examples of how pharmacists could convey that message. This study demonstrates the importance of qualitative work in this field, which can highlight the significance of listening to patients' voices and stresses the importance for pharmacists to take care of each subject who struggles with addiction at an individual level. In particular, the value of a pharmacist-patient relationship and effective communication were highlighted.

PLWHA experience stigma, mental health conditions, and addiction at higher rates than the general U.S. population.^20^^,^^21^ As such, they are more often marginalized or outright ostracized from society, their communities, or individual families.^21^^,^^22^Our findings suggest that the divorce of these patients from traditional sources of support fosters a crucial role for the pharmacist. Trust, rapport, and overall patient relationships may indeed be easier, and not more difficult as some might believe,^8^ to establish for this group who experiences stigma both through the virtue of their HIV diagnosis and opioid misuse. This finding is particularly interesting when contrasting it with a survey conducted among Tennessee community pharmacists, who felt that they lack confidence in discussing substance misuse and consequently will not change patient behavior.^8^ In the present study, patients not only valued such counseling but also indicated a desire for additional and more assertive communication from their pharmacist. This concept is of particular importance for the community pharmacist who is often seen as an important member of the community, is easily accessible, and by these virtues tends to foster strong personal relationships. Further research is needed to better elucidate the role of the pharmacist for stigmatized populations and how best to approach these patients in practice.

Results also indicated that pharmacist-patient interactions positively impact adherence to ARV medications. Subjects felt that they were generally adherent to the ARV regimen; however, a few highlighted various insurance-related obstacles that the pharmacist solved. Note, this role is often overlooked and under emphasized in clinical guidelines and research studies in general; however, as our study demonstrates, the unique skill of the pharmacist to use their clinical knowledge to navigate the complex drug formularies and tiered drug coverage categories among the various third-party payers has downstream impacts on both clinical and humanistic outcomes. Similar findings were reflected in the experiences of patients with pharmacists in other studies.^23^^,^^24^ Several subjects in this study mentioned that addiction had a negative impact on their life and adherence to ARV regimen because they would look for any opportunity to obtain pain medications from other resources. This finding is consonant with existing literature.^25^^,^^26^

These findings add to the literature of the pharmacist's growing positive impact on public health. This underutilized role in public health has been emphasized during the COVID-19 pandemic. Although to many the “corner druggist” is still viewed as solely a distributor of medicines on order from a physician, it is evident to the broader U.S. population that the pharmacists serve as a convenient, quality healthcare destination.^27^^,^^28^ In terms of treatment and testing, pharmacies continue to add to their menu of health services available – opening the door to newcomers to the healthcare system as well as offering a more convenient location for some services for those already engaged.^29^^,^^30^ In the present study, we see a new patient population who may be served by pharmacists – those stigmatized because of their demographics and chronic health conditions. To date, little has been investigated in the way of the pharmacist's public health role for these patients beyond traditional medication distribution services. However, in light of these findings, there is a need for further research on expanding care to these underserved communities with the help of pharmacists – especially in leveraging preestablished, strong relationships between the community pharmacist and their patient population.

In similar vein, there is a need to better understand the complexities of patient-pharmacist relationship for this patient population. It would be understandable for patients with stigmatized conditions, including HIV and substance use disorder, to withhold trust from any medical professional. However, we instead see in this study a willingness to both trust in and accept a relationship with the pharmacist. A recent systematic review of research on patient-provider relationships noted that the depth of the relationship was formed through both perceptions of the direct experiences with the provider and time spent under the provider's care.^23^ This framework fits the findings of the present study given the general positive experiences the study subjects had with the pharmacist as well as the fact that patients encounter pharmacists on average more than their primary care physician.^24^ Importantly, it is not only the patient's perception of the pharmacist which contributes to the relationship, but also the patient's perception of the pharmacist's opinion of the patient. Given the qualitative nature of the data here, it is not possible to make definite statements about patient perceptions of pharmacists globally; however, it would stand to reason that these findings would be similar for those pharmacists who provide the patient with a positive experience consistently and show outwardly their support of individuals who often experience stigma. There are important lessons here both for academic researchers and educators in training students and practitioners on their approach to patients generally, and more specifically for those who are stigmatized.

## 5 Strengths, limitations, and clinical implications

The results of this study should be interpreted in the light of some limitations. Due to the study design, the findings cannot be generalized to all PLWHA and OUD in Tennessee. Additionally, subjects self-identified as opioid and ARV users; however, no medical records were consulted. The subjects learned about this study via fliers located in HIV clinics and centers situated in distinct geographic regions of Tennessee. Consequently, the sample was heterogeneous from the race, ethnicity, and educational background. Future studies may focus on recruitment solely from HIV clinics where medical records could confirm the medication regimens.

It is important to note that every patient-pharmacist interaction, especially with stigmatized and underserved patients, is different and unique, and these interactions are crucial. Community pharmacists could benefit from continuing education credits that focus on counseling and interacting with PLWHA who are using opioid medications. When interacting with PLWHA who are taking opioid prescriptions, the community pharmacist could use a private space to facilitate privacy and initiate a trustful relationship. After assuring the patient that the information about their health conditions and medications is confidential, the pharmacist could focus on educating about opioid misuse and the consequences of abusing the opioid medications. As vital members of the interdisciplinary care for patients, community pharmacists could also use their working relationship with prescribing providers to be able to discuss patient concerns as they arise to improve patient outcomes.

Since this study is a limited cohort from Tennessee, it is imperative to expand this study using large cohort of subjects from different demographic, gender, race, and ethnic groups. A multitude of data obtained from a large study would provide a nationwide perspective on the important role pharmacists play in counseling and effectively managing patients' health.

## 6 Conclusion

This study presented findings from a TPB analysis of the PLWHA experiences with pharmacists and usage of ARV and opioid medications. This study described two key themes that emerged from this qualitative study that highlight the importance of developing relationships with PLWHA. The data highlights the accessibility, convenience, and the relationship developed with pharmacists. These results depicted how some subjects are using the pharmacists as a key resource for medication information. These findings also demonstrated how for some subjects a more detailed counseling session when they receive opioid medications could be crucial in changing their behavior related to opioid use as this may result in reduced use, but more importantly, reduced misuse. Thus, community pharmacists are well positioned to reduce the usage of opioid medications and change PLWHA behavior and attitudes toward opioid prescriptions. Thus, this study can act as a catalyst to future studies on the experiences of PLWHA and OUD to understand how they want to be counseled by pharmacists.

## Funding

UTHSC College of Pharmacy Seed Fund.

## Author contribution

AC, MPV, and SK conceptualized the study. AC collected the data and analyzed it. KH and MPV reviewed the analyzed data. NAS placed fliers in the HIV clinic. AC, MPV, KH, and NAS wrote the first draft of the manuscript. All the authors reviewed and agreed to the current version of the manuscript.

## Declaration of Competing Interest

The authors declare that they have no known competing financial interests or personal relationships that could have appeared to influence the work reported in this paper.

## References

[1] Schafer J.J., Gill T.K., Sherman E.M., McNicholl I.R., Hawkins B. (2016). Ashp guidelines on pharmacist involvement in hiv care. Am J Health Syst Pharm.

[2] Schaecher K.L. (2013). The importance of treatment adherence in hiv. Am J Manag Care.

[3] Cernasev A., Veve M.P., Cory T.J. (2020). Opioid use disorders in people living with hiv/aids: a review of implications for patient outcomes, drug interactions, and neurocognitive disorders. Pharmacy..

[4] Cernasev A., Kodidela S., Veve M.P., Cory T., Jasmin H., Kumar S. (2021). A narrative systematic literature review: a focus on qualitative studies on hiv and medication-assisted therapy in the United States. Pharmacy..

[5] Peters P.J., Pontones P., Hoover K.W. (2016). Hiv infection linked to injection use of oxymorphone in Indiana, 2014–2015. N Engl J Med.

[6] Bach P., Hartung D. (2019). Leveraging the role of community pharmacists in the prevention, surveillance, and treatment of opioid use disorders. Addict Sci Clin Pract.

[7] Kibicho J., Pinkerton S.D., Owczarzak J., Mkandawire-Valhmu L., Kako P.M. (2015). Are community-based pharmacists underused in the care of persons living with hiv? A need for structural and policy changes. J Am Pharm Assoc.

[8] Hagemeier N.E., Murawski M.M., Lopez N.C., Alamian A., Pack R.P. (2014). Theoretical exploration of Tennessee community pharmacists’ perceptions regarding opioid pain reliever abuse communication. Res Soc Adm Pharm.

[9] Ajzen I. (1991). The theory of planned behavior. Organ Behav Hum Decis Process.

[10] Siuki H.A., Peyman N., Vahedian-Shahroodi M., Gholian-Aval M., Tehrani H. (2019). Health education intervention on hiv/aids prevention behaviors among health volunteers in healthcare centers: an applying the theory of planned behavior. J Soc Serv Res.

[11] Norman P., Conner M., Bell R. (1999). The theory of planned behavior and smoking cessation. Health Psychol.

[12] Quintana-Bárcena P., Lalonde L., Lauzier S. (2019). Beliefs influencing community pharmacists’ interventions with chronic kidney disease patients: a theory-based qualitative study. Res Soc Adm Pharm.

[13] Malo T.L., Hall M.E., Brewer N.T., Lathren C.R., Gilkey M.B. (2018). Why is announcement training more effective than conversation training for introducing hpv vaccination? A theory-based investigation. Implement Sci.

[14] Braun V., Clarke V. (2006). Using thematic analysis in psychology. Qual Res Psychol.

[15] Lincoln Y.S., Guba E.G. (1985).

[16] Saunders B., Sim J., Kingstone T. (2018). Saturation in qualitative research: exploring its conceptualization and operationalization. Qual Quant.

[17] Morse J.M. (2004). Theoretical saturation. Encycl Social Sci Res Methods.

[18] Tong A., Sainsbury P., Craig J. (2007). Consolidated criteria for reporting qualitative research (coreq): A 32-item checklist for interviews and focus groups. Int J Qual Health Care.

[19] (HRSA) HRSA About the ryan white hiv/aids program. https://hab.hrsa.gov/about-ryan-white-hivaids-program/about-ryan-white-hivaids-program.

[20] Weaver M.R., Conover C.J., Proescholdbell R.J., Arno P.S., Ang A., Ettner S.L. (2008). Utilization of mental health and substance abuse care for people living with hiv/aids, chronic mental illness, and substance abuse disorders. JAIDS.

[21] Turan B., Budhwani H., Fazeli P.L. (2017). How does stigma affect people living with hiv? The mediating roles of internalized and anticipated hiv stigma in the effects of perceived community stigma on health and psychosocial outcomes. AIDS Behav.

[22] Duffy L. (2005). Suffering, shame, and silence: the stigma of hiv/aids. J Assoc Nurses AIDS Care.

[23] Ridd M., Shaw A., Lewis G., Salisbury C. (2009). The patient–doctor relationship: a synthesis of the qualitative literature on patients’ perspectives. Br J Gen Pract.

[24] Berenbrok L.A., Gabriel N., Coley K.C., Hernandez I. (2020). Evaluation of frequency of encounters with primary care physicians vs visits to community pharmacies among medicare beneficiaries. JAMA Netw Open.

[25] Biancarelli D.L., Biello K.B., Childs E. (2019). Strategies used by people who inject drugs to avoid stigma in healthcare settings. Drug Alcohol Depend.

[26] Stringer K.L., Marotta P., Baker E. (2019). Substance use stigma and antiretroviral therapy adherence among a drug-using population living with hiv. AIDS Patient Care STDs.

[27] Cadogan C.A., Hughes C.M. (2021). On the frontline against covid-19: community pharmacists’ contribution during a public health crisis. Res Soc Adm Pharm.

[28] Hussain R., Dawoud D.M. (2021). Drive-thru pharmacy services: a way forward to combat covid-19 pandemic. Res Soc Adm Pharm.

[29] Klepser M.E., Klepser D.G., Dering-Anderson A.M., Morse J.A., Smith J.K., Klepser S.A. (2016). Effectiveness of a pharmacist-physician collaborative program to manage influenza-like illness. J Am Pharma Assoc.

[30] Laws M., Scott M.K. (2008). The emergence of retail-based clinics in the United States: early observations. Health Aff.

